# Game-Related Volleyball Skills that Influence Victory

**DOI:** 10.2478/hukin-2014-0045

**Published:** 2014-07-08

**Authors:** Miguel Silva, Daniel Lacerda, Paulo Vicente João

**Affiliations:** 1 Department of Sports Sciences, University of Trás-os-Montes and Alto Douro (UTAD), Vila Real - Portugal.; 2 CIDESD - UTAD - Research Unit, Sport Sciences, Exercise and Health Department, University of Trás-os-Montes and Alto Douro, Vila Real - Portugal.

**Keywords:** volleyball, games-related statistics, performance, results, skills

## Abstract

The aim of the present study was to identify the volleyball skills that discriminate in favour of victory. Twenty-four games (n=24) from the Senior Men’s Volleyball World Championship played in Italy in 2010 were chosen and analyzed with Data Volley software. The discriminating function was used to identify the discriminating variables, using a canonical structuring coefficient of |SC| ≥ .30. The results suggest that service points, reception errors, and blocking errors were the discriminating variables that identify the final outcome of the match (victory/defeat). Moreover, successful service points were the major variable most likely associated with match success (victory). In this sense, increasing the effectiveness of service should be a top priority in coaching elite volleyball teams.

## Introduction

The evolution of training in volleyball has been reflected in the increased homogeneity of high-level athletes’ characteristics (Sheppard et al., 2009). Top teams are similar in average body height, and in physical and technical performances (Gabbett et al., 2007). Hence, matches between the best teams are often very balanced. Understanding how the skill performance indicators relate to scoring of points is useful for athletes and coaches in all team sports (Lobietti, et al., 2010; Marcelino et al., 2010; Miskin et al., 2010; Palao et al., 2005; Zetou et al., 2007). As most of these teams, if not all, are subject to highly specialized supervision in several fields such as medical examination, physical preparation (Golik-Peric et al., 2011; Trajkovic et al., 2012), psychological support, diet control (Valliant et al., 2012) and tactical orientation (João et al., 2010; Palao et al., 2005), it is important to know which skills in volleyball contribute most to victory.

When examining the different skills performance on display in a volleyball match (serving, blocking, attacking, reception, setting, and defense), it seems reasonable that the team that makes the fewest errors should be the one that is most likely to succeed. The results observed by Castro et al. (2011) and Drikos et al. (2009) revealed a significant influence of serve and attack efficacy (those that result in direct point) on the match outcome.

Attacks, blocks, and serves, due to the possibility of scoring a direct point, are considered Scoring Skills (Marcelino et al., 2010). On the other hand, the defense, setting, and reception procedures are termed Non Scoring Skills (Marcelino et al., 2010) and therefore should, at first glance, contribute less to a win. Despite this classification it is important to acknowledge those skills that most discriminate in favour of victory. Moreover, these analyses have been mostly based on the relation between efficacy and success. However, in some studies (Bergeles, et al., 2009; Buscà and Febrer, 2012) it has been proven that in elite teams, error in some skills may indicate a higher level of risks taken rather than technical problems.

While recognizing the significance of information from previous research on this topic, we could notice a lack of studies in volleyball that focus on the analysis of performance factors distinguishing winning teams from losing ones. Therefore, the aim of the present study was to identify the skills that discriminate in favour of victory. It seems appropriate to conduct this research in order to improve training methodologies and performance in competition, providing useful indicators for coaches, players and their teams.

## Material and Methods

The sample in this study was composed of 24 matches (n=24) from the 2010 FIVB Men’s World Championships in Italy, with a total of 90 sets played; 24670 separate actions were analyzed: 4083 services, 3434 receptions, 4906 attacks (3030 from side-out and 1876 from counter attack), 2109 blocks, 1933 digs, and 3299 sets. The data were collected and analyzed with Data Volley software.

This sample was chosen because it consisted of high-level matches from the last World Championship. To ensure that the analysis focused on balanced high-level matches, the matches of the 12 best teams (3rd phase) were chosen, ending with the match to award the first and second places (final).

### Measures

The dependent variables considered were the result of the match (victory and defeat). The independent variables were chosen in accordance with suggestions by other authors, including terminal actions such as serves, blocks, and side out attacks (complex I, composed by reception, setting and attack) and attacks in counter attack (complex II, constituted by defense, setting and attack) (João et al., 2010; Marelic et al., 2004; Palao et al., 2004).

Due to the fact that several authors (João et al., 2006; Maia and Mesquita, 2006; Palao et al., 2004) have referred to reception, setting, and defense as essential factors in the development of a match, we also included these variables in our research design.

This study considered only actions that could be determined to be absolute successes or failures, since they may be more easily associated with the final outcome. We omitted continuity skills because they do not reflect the mastery of a particular skill, but rather a set of skills.

Side-out (or complex I), reception, setting, and attack.

Counter-attack (or complex II), service, block, dig, setting, and attack.

### Procedures / Data collection

The data were collected using Data Volley software. By applying this software it is possible to display and print at any time analytical reports detailing any and all information needed to objectively evaluate the performance of the team. This program allows access to total and by set detailed qualitative statistics, offering a wealth of data for researchers and coaches. Twenty-two of the 24 teams present at the competition used this software, what confirms the utility and validity of the information provided by the software.

### Reliability

The reliability of the observations was tested, presenting intra-observer Cohen’s Kappa values between 0.96 and 1, and Cohen’s Kappa inter-observer values of 0.98 and 1, which meant that the data were reliable. Reliability analysis of the data was carried out with the SPSS (18), using a significance degree of 5%.

### Statistical Analysis

To identify which variables discriminated by result, a discriminant analysis was computed, using a coefficient structure superior of |SC| ≥.30 (Tabachnick and Fidell, 1996) to determine the indicators that contributed most to differences between victories and defeats. Corresponding effect sizes (Hedge’s g and 95% confidence intervals) were also calculated to evaluate the magnitude of the statistically significant differences. All data were analyzed with the statistical package SPSS for Windows, release 18.0 (SPSS, Inc., Chicago, IL). Effect sizes were assessed with Comprehensive Meta-Analysis (Version 2.0) and statistical significance was set at 5%.

## Results

The means and the standard deviations of game-related statistics are presented in Table 2. The discriminant analysis yielded statistically significant differences (p<.05) between victories and defeats. Only the side-out error variable (g=1.33) presents significant differences that are possible to be analyzed with this effect size.

As seen in Table 3, the average vector that contributed most to discrimination between defeat and victory was composed of the serve point (SC=0.42), reception errors (SC= −0.35) and blocking errors (SC=0.32). The remaining variables did not show significant contributions to the structure of the linear function. The obtained function presents a statistically significant difference χ^2^ = 25.143, p≤.05, with a canonical correlation of .96.

## Discussion

The main results of this study showed that the skills that discriminate in favour of victory are the serve point and surprisingly, blocking errors. On the other side, reception errors were the only variable that discriminated in favour of defeat.

Our results clearly point to the importance of the serve point in determining victory. This result becomes even more relevant when we pay attention to the value of its effect size (−1.10), which is quite high and allows for generalization of results. Because the teams are evenly balanced, when a match gets close to the end, this skill (serving) may be associated with victory. Our results confirm the observations of Zetou et al. (2007), who mention that the ace (direct serve) is a predictor of victory in high performance teams.

In accordance with Marelic et al. (2004), the team that serves better has a tendency to win more sets. A more attentive analysis of the results highlights the fact that the number of serves that result in direct points was very low, but higher in the winning teams. Serve errors were also less frequent in winning teams.

In fact, some researchers believe that teams that are at a disadvantage in the set take more risks while serving, probably because they have nothing to lose (João et al., 2010; Marelic et al., 2004). By risking more strategically, these teams also end up failing more frequently, consequently increasing the percentage of errors made (Marelic et al., 2004; Yiannis et al., 2004). On the other hand, if the serve is risky, opponent reception will be more difficult, increasing error probability. Our results discriminate reception error in defeat so the teams with low efficacy in this skill are more likely to lose the game.

It is important, therefore, to increase the efficacy of the serve, since it is considered a terminal action (Marelic et al., 2004), and may result in a direct point. In that sense, we can infer that the serve is of crucial importance in the performance of volleyball teams. The importance of practicing this skill in the training process is quite clear.

Literature regarding blocking skills in volleyball pointed to its importance for the match outcome (Afonso et al., 2010; Palao et al., 2004). Surprisingly, our results revealed that blocking errors discriminate in favour of victory. Errors in blocking may result in one of the following three situations: (1) point for the opposing team, (2) continuity of the match by the team itself (if the defense is good), or (3) continuity for the opposing team. According to these possibilities our results suggest that in high level balanced volleyball teams blocking errors result in more frequent continuity situations than in scoring points.

The organization of the opposition’s first line of defense, through strategies and triple block formations, may increase the probability of successful blocking. This fact may also be a consequence of the speed of the ball, the variability of the setting, and the trajectory, making it difficult to effectively organize blocks (Afonso et al., 2005; Zetou et al., 2007). The diversity of results that may arise when this skill is employed may explain why studies of blocking errors have yielded mixed results. A thorough analysis of our results shows that the winning teams made more blocks (block points and block errors).

Palao (2008) concluded that successful blocking offers more chances to win. In addition, the block is the first terminal action that the opposition may take to the opponent’s attack, and may result in a direct point.

Regarding reception errors, our results suggest that this factor, as would be expected, may be associated with defeat. Several studies have verified a positive association between efficacy in reception and the final result of the match (João et al., 2006; Laios and Kountouris, 2005; Maia and Mesquita, 2006). Even though reception is not a terminal action, a perfect reception allows the setter to organize the team offensively with all the possibilities of attack, increasing the probability of winning the match (João et al., 2010). In elite teams, like the ones analyzed in the present study, the receiving players are very experienced, so only errors in reception discriminated for result.

In conclusion, as the world’s top teams continue to become more similar and balanced, competition must be evaluated in terms of performance details. Some skills are more important than others as they are associated with success; while poor performance in other skills leads to failure. In order to improve performance, coaches must prepare their teams, evaluate the opponent, and focus on the skills that may discriminate in favour of victory and improve the factors that result in failure. Our results highlight the importance of serving successfully, improving blocking continuity situations, as well as, minimizing errors in reception.

The results of this study of the last World Championship confirm that an effective serve is a variable that may be used to predict success. Therefore, serve training is crucial, and should be taken in consideration in different contexts and moments during the match, using several types of strategies and scenarios that may cause imbalances between teams at the same sports level.

The study’s most interesting finding is that errors are also associated with victory. In fact, blocking errors discriminated in favour of victory, but winning teams had a higher percentage of successful blocks. Blocking continuity situations should also play an important role in training concepts.

Finally, reception errors discriminated in favour of defeat, which highlights the importance of practicing this skill to avoid failure.

## Figures and Tables

**Table 1:** Game related Skills description

Skills	Description
Service error	Error occurred in the service
Serve point	Efficacy with service
Reception error	Error occurred in reception
Excellent reception	Efficacy in reception
Attack error	Error occurred in attack
Attack point	Efficacy with attack
Side out error	Error reception, set and attack
Side out point	Efficacy with reception, set and attack
Counter attack error	Error after defense, set and attack
Counter attacker point	Efficacy after defense, set and attack
Blocking error	Error occurred in the block
Blocking point	Efficacy with block
Dig error	Error occurred in the defense
Excellent dig	Efficacy in defense
Set error	Error occurred in the sets
Set excellent	Efficacy with a sets

**Table 2:** Means and standard deviations of game-related statistics by result (victory or defeat), effect size and 95% CI.

Game related statistics	n	Defeat	n	victory	F	*p*	*Effect Size*	CI 95%
		M ± *Dp*		M ± *Dp*			*(Hedges’s g)*	
Service error	951	13.00 ± 3.52	1093	16.25 ± 4.27	2.29	.156	−0.82	−0.91, −0.73
Serve point	72	2.67 ± 1.51	92	4.75 ± 2.12	4.17	.064	−1.10	−1.43, −0.77
Reception error	325	3.83 ± 2.23	294	4.13 ± 2.17	0.06	.810	−0.13	−0.29, 0.02
Excellent reception	565	27.00 ± 7.07	522	23.88 ± 9.70	0.44	.519	0.37	0.25, 0.49
Attack error	609	12.50 ± 3.02	566	8.88 ± 3.98	3.46	.088	1.02	0.90, 1.15
Attack point	1113	44.00 ± 11.37	1237	54.25 ± 11.96	2.62	.131	−0.87	−0.96, −0.79
Side-out error	352	8.67 ± 2.73	291	5.13 ± 2.53	628	.028^*^	1.33	1.16, 1.51
Side-out point	758	29.83 ± 6.08	794	34.50 ±10.25	0.97	.343	−0.55	−0.65, −0.45
Counter Attack error	89	3.83 ± 2.04	443	3.75 ± 2.05	0.01	.941	0.03	−0.18, 0.26
Counter Attack point	248	14.17 ± 5.67	275	19.75 ± 5.60	3.37	.091	−0.98	−1.17, −0.80
Blocking error	69	16.50 ± 6.25	94	15.25 ± 3.11	0.24	.630	0.26	−0.04, 0.57
Blocking point	173	8.67 ± 3.20	273	12.25 ± 3.77	3.50	.086	−1.00	−1.20, −0.80
Dig error	42	13.50 ± 6.77	80	13.50 ± 3.21	0.00	1.000	0.00	−0.37, 0.37
Excellent Dig	320	15.17 ± 7.33	323	10.75 ± 6.18	1.50	.245	0.65	0.49, 0.81
Set error	40	1.83 ± 0.75	18	1.75 ± 1.16	0.02	.882	0.08	−0.46, 0.64
Set excellent	1368	21.33 ± 6.71	1302	20.13 ± 7.32	0.10	.757	0.17	0.09, 0.24

M: means; SD: standard deviation; f: ratios; g: Hedges’s g; CI 95% confidence intervals.

* p<.05

**Table 3:** Discriminant function structure coefficients and tests of statistical significance.

Game related statistics	SC
Serve point	.42^*^
Reception error	−.35^*^
Blocking error	.32^*^
Side-Out error	−.25
Blocking point	.15
Attack error	−.15
Service error	.12
Excellent dig	−.10
Dig error	.07
Set excellent	.07
Counter Attack error	.06
Excellent reception	−.05
Attack point	.03
Counter Attack point	.03
Side-Out point	.02
Set error	.02

Wilks’ Lambda	.07
Eigenvalue	13.1
Canonical correlation	.96

* |SC| ≥ .30.

## References

[b1-jhk-41-173] Afonso J, Mesquita I, Palao J (2005). Relationship between the use of commit-block and the numbers of blockers and the block effectiveness. Int J Perform Anal Sport.

[b2-jhk-41-173] Afonso J, Mesquita I, Marcelino R, Silva J (2010). Analysis of the setter’s tactical action in high-performance women’s volleyball. Kinesiology.

[b3-jhk-41-173] Bergeles N, Barzouka K, Nikolaidou M (2009). Performance of male and female setters and attackers on Olympic-level Volleyball teams. Int J Perform Anal Sport.

[b4-jhk-41-173] Buscà B, Febrer J (2012). Temporal fight between the middle blocker and the setter in high level volleyball. Rev. int. med. cienc. act. fís. deporte.

[b5-jhk-41-173] Castro J, Souza A, Mesquita I (2011). Attack efficacy in volleyball: Elite male teams. Percept motor skill.

[b6-jhk-41-173] Drikos S, Kountouris P, Laios A, Laios Y (2009). Correlates of Team Performance in Volleyball. Int J Perform Anal Sport.

[b7-jhk-41-173] Gabbett T, Boris G, Nathan D (2007). The use of physiological, anthropometric, and skill data to predict selection in a talent-identified junior volleyball squad. J Sport Sci.

[b8-jhk-41-173] Golik-Peric D, Drapsin M, Obradovic B (2011). Short-Term Isokinetic Training Versus Isotonic Training: Effects on Asymmetry in Strength of Thigh Muscles. J Hum Kinet.

[b9-jhk-41-173] João P, Leite N, Mesquita I, Sampaio J (2010). Sex differences in discriminative power of volleyball game-related statistics. Percept motor skill.

[b10-jhk-41-173] João P, Mesquita I, Sampaio J, Moutinho C (2006). Comparative analysis between libero and priority receivers on the offensive organization, from the serve reception on the volleyball game. Rev Port Cien Desp.

[b11-jhk-41-173] Laios Y, Koutouris P (2005). Evolution in men’s volleyball skills and tactics as evidenced in the Athens 2004 Olympic Games. Int J Perform Anal Sport.

[b12-jhk-41-173] Lobietti R, Coleman S, Pizzichillo E, Merni F (2010). Landing techniques in volleyball. J Sport Sci.

[b13-jhk-41-173] Maia N, Mesquita I (2006). Study of zones and efficacy of the reception according the receiver player in female senior volleyball. Rev. bras. educ. fís. esporte.

[b14-jhk-41-173] Marcelino R, Mesquita I, Sampaio J, Moraes J (2010). Study of performance indicators in male volleyball according to the set results. Rev. bras. educ. fís. esporte.

[b15-jhk-41-173] Marelic N, Resetar T, Jankovic V (2004). Discriminant analysis of the sets won and the sets lost by one team in a1 Italian volleyball league – a case study. Kinesiology.

[b16-jhk-41-173] Miskin M, Fellingham G, Florence L (2010). Skill Importance in Women’s Volleyball. J. Quant. Anal. Sports.

[b17-jhk-41-173] Palao J (2008). Options for analysis of the volleyball score sheet. Int J Perform Anal Sport.

[b18-jhk-41-173] Palao J, Santos J, Ureña A (2005). Effect of setter’s position on the spike in volleyball. J hum movement stud.

[b19-jhk-41-173] Palao J, Santos J, Ureña A (2004). Effect of team level on skill performance in volleyball. Int J Perform Anal Sport.

[b20-jhk-41-173] Sheppard J, Gabbett, Tim J, Stanganelli L (2009). An Analysis of Playing Positions in Elite Men’s Volleyball: Considerations for Competition Demands and Physiologic Characteristics. J Stren Cond Res.

[b21-jhk-41-173] Tabachnick B, Fidell L (1996). Using Multivariate Statistics.

[b22-jhk-41-173] Trajkovic N, Milanovic Z, Sporis G, Milic V, Stankovic R (2012). The effects of 6 weeks of preseason skill-based conditioning on physical performance in male volleyball players. J Stren Cond Res.

[b23-jhk-41-173] Valliant M, Emplaincourt H, Wenzel R, Garner B (2012). Nutrition Education by a Registered Dietitian Improves Dietary Intake and Nutrition Knowledge of a NCAA Female Volleyball Team. Nutrients.

[b24-jhk-41-173] Yiannis L, Panagiotis K, Ioannis A, Alkinoi K (2004). A comparative Study of the Effectiveness of the Greek national Men’s Volleyball Team with Internationally Top-Ranked Teams. International Journal of Volleyball Research.

[b25-jhk-41-173] Zetou E, Moustakidis A, Tsigilis N, Komninakidou A (2007). Does Effectiveness of Skill in Complex I Predict Win in Men’s Olympic Volleyball Games?. J. Quant. Anal. Sports.

